# A higher order visual neuron tuned to the spatial amplitude spectra of natural scenes

**DOI:** 10.1038/ncomms9522

**Published:** 2015-10-06

**Authors:** Olga Dyakova, Yu-Jen Lee, Kit D. Longden, Valerij G. Kiselev, Karin Nordström

**Affiliations:** 1 Department of Neuroscience, Uppsala University, Box 593, 75124 Uppsala, Sweden; 2 HHMI Janelia Research Campus, 19700 Helix Drive, Ashburn, Virginia 20176, USA; 3 Medical Physics, Department of Radiology, University Medical Center Freiburg, Breisacher Strasse 60a, 79106 Freiburg, Germany; 4 Anatomy and Histology, Centre for Neuroscience, Flinders University, GPO Box 2100, Adelaide, South Australia 5001, Australia

## Abstract

Animal sensory systems are optimally adapted to those features typically encountered in natural surrounds, thus allowing neurons with limited bandwidth to encode challengingly large input ranges. Natural scenes are not random, and peripheral visual systems in vertebrates and insects have evolved to respond efficiently to their typical spatial statistics. The mammalian visual cortex is also tuned to natural spatial statistics, but less is known about coding in higher order neurons in insects. To redress this we here record intracellularly from a higher order visual neuron in the hoverfly. We show that the cSIFE neuron, which is inhibited by stationary images, is maximally inhibited when the slope constant of the amplitude spectrum is close to the mean in natural scenes. The behavioural optomotor response is also strongest to images with naturalistic image statistics. Our results thus reveal a close coupling between the inherent statistics of natural scenes and higher order visual processing in insects.

## Abstract (web summary)

Natural scenes contain statistics that are constrained in both space and time. Here, the authors show that, in insects as in mammals, both higher order neural mechanisms and behavioural discrimination are tuned to natural spatial statistics.

## Graphical abstract

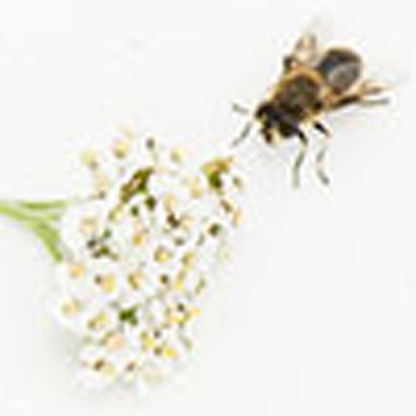

A major challenge of animal sensory systems is to appropriately encode incoming stimuli that vary enormously, while using noisy neuronal signalling with limited bandwidth. The natural input that biological visual systems encounter is not random, but contains statistics that are remarkably constrained in both space and time^1^,^2^. Photographs of natural scenes can be statistically analysed using the Fourier transform^3^,^4^,^5^,^6^ that describes the image as a set of spatial frequencies of given amplitudes and phases, and different orientations. The phase spectrum is linked to the characteristic contours, edges and features that together identify the unique structure of a particular image^6^,^7^. Consequently, if the phase spectrum is disturbed, the human observer perceives the new image as completely unrecognizable^8^.

The amplitude spectrum of a natural scene tends to follow a power law^1^,^5^:


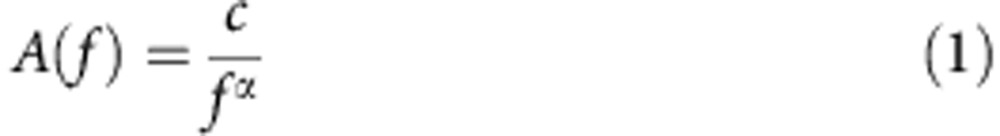


in which the amplitude of a given frequency, *A(f)*, is inversely proportional to spatial frequency (*f*) raised to the power *α*. Because *α* is readily apparent as the slope of the log–log plot of the amplitude spectrum, it is referred to as the slope constant. If the slope constant of an image is increased, the human observer perceives this as the image getting blurrier, but the scene itself remains recognizable^4^. In natural scenes, the amplitude spectra of spatial frequencies differ according to orientation. Horizontal and vertical structures, such as the horizon and tree trunks, increase amplitudes at horizontal and vertical orientations^9^,^10^, and this effect is more pronounced in built environments^11^.

In images where α is exactly 1, the image is scale invariant, which means that it has the same amount of detail regardless of viewing scale^3^. In other words, an image with a perfect 1/*f* amplitude spectrum has equal amounts of energy in each octave (for example, 1–2, 2–4, 4–8, 8–16 cycles per degree, c.p.d^4^). In practice, the slope constants vary across scenes, and published slope constants show a broad Gaussian distribution with a peak around 1–1.2 (refs 3, 12, 13). However, it has been suggested that the broad spread could be an artefact of the varying densities of objects and textures subtending small angles and large angles across scenes^4^.

Since animal vision has evolved in natural scenes with largely predictable statistics, it is well established that the role of peripheral vision is to reduce the predictable redundancy^14^,^15^. For example, low temporal and spatial frequencies predominate naturalistic visual input^12^,^16^. In both mammals and insects low-frequency redundancy is suppressed via lateral inhibition and temporal antagonism^17^,^18^,^19^,^20^ in photoreceptors and associated peripheral neurons. Consequently, retinal filters in fly lamina monopolar cells (LMCs, the first interneurons in the insect visual pathway) and ganglion cells of the vertebrate retina ‘whiten' the signal^2^,^16^,^19^,^20^.

In mammals, higher order processing of naturalistic input has typically been investigated using psychophysics^5^, functional magnetic resonance imaging^21^ or modelling^22^,^23^. Such analyses show that the mammalian visual cortex is optimally tuned to the spatial statistics of natural scenes. Most insect data come from the analysis of peripheral visual processing (for example, photoreceptors or LMCs^17^,^18^,^19^), or using naturalistic stimuli that also vary in time^24^,^25^. Here, we quantify how the response of an insect higher order visual neuron depends on the strictly spatial characteristics of natural scenes. For this, we utilize a recently described neuron that is excited by flicker, and thus responds non-directionally to motion, and more valid for our purposes here, is inhibited by stationary images^26^. The inhibition of centrifugal Stationary Inhibited Flicker Excited, cSIFE, by stationary images provides a unique opportunity for investigating visual responses to natural scenes that vary only in the spatial domain, while remaining constant in the temporal domain. We record intracellular responses of cSIFE and show that the response inhibition to natural scenes depends strongly on the slope constant (α in Eq. 1). Indeed, we find a peak inhibition when the slope constant is close to 1, that is, close to those most prevalent in natural scenes^1^,^3^. We further show that the behavioural optomotor response depends on the slope constant, and find that this is strongest when α is close to 1. Our data thus show that in insects, as in mammals^5^,^21^, both higher order neural mechanisms and behavioural discrimination are tuned to natural spatial statistics.

## Results

### cSIFE is inhibited by stationary natural scenes

**Figure 1:**
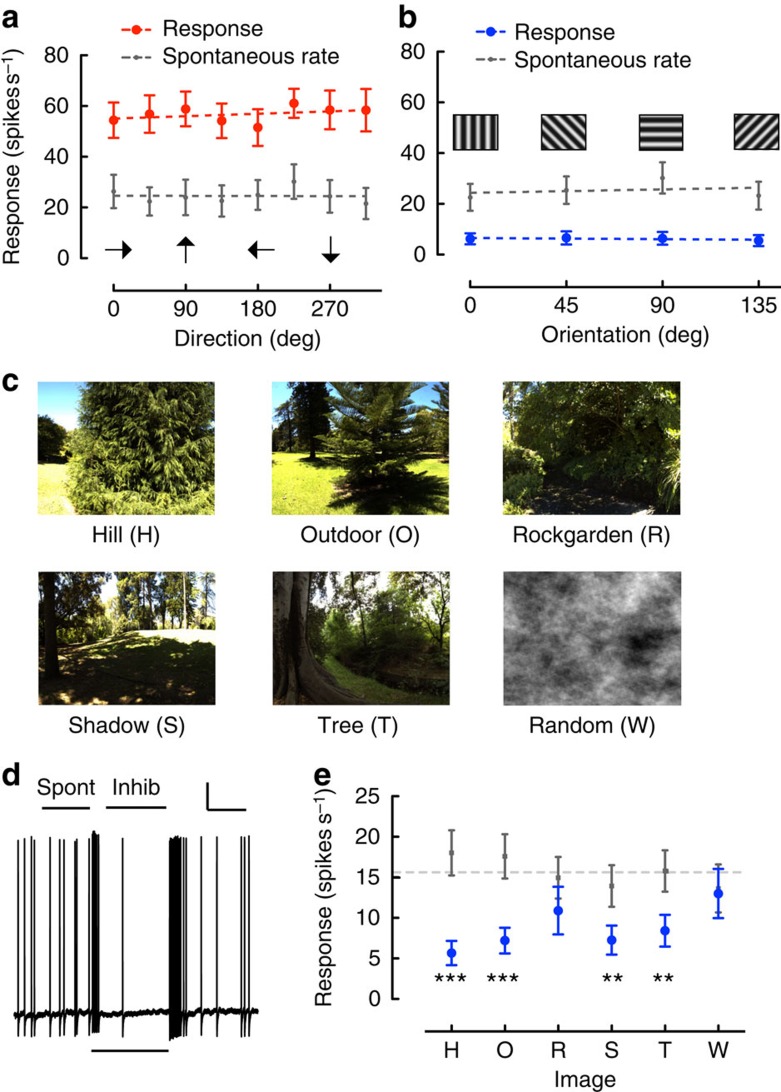
cSIFE is inhibited by stationary natural images. (**a**) cSIFE is excited by sinusoidal gratings moving at 5 Hz (8° wavelength) regardless of the direction of motion. Response in red and spontaneous rate in grey, error bars show s.e.m., *N*=16. (**b**) cSIFE is inhibited by the same sinusoidal gratings when stationary, regardless of their orientation. Inhibition in blue, and spontaneous rate in grey, error bars show s.e.m., same *N*=16. (**c**) The Hill, Outdoor, Rockgarden, Shadow, Tree images^25^,^28^,^29^, and a filtered Random image^30^. The luminance and contrast of the images have been rescaled for better printing. (**d**) cSIFE's inhibition induced by a stationary natural image, with the peri-stimulus duration (1 s) indicated with a bar under the raw data. ‘Spont' and ‘inhib' show the analysis windows used in the rest of the paper. The scale bar shows 10 mV and 100 ms. (**e**) The cSIFE response to the six scenes in **d**. Inhibition in blue and spontaneous rate in grey, error bars indicate s.e.m., *N*=16. The dashed line shows the average spontaneous rate. Stars (*) indicate significant difference between the inhibition and the spontaneous rate (two-way ANOVA followed by Bonferroni's multiple comparison test, **P<0.01, and ***P<0.001).

cSIFE is a higher order neuron of the hoverfly lobula plate. As opposed to the more well-studied classic lobula plate tangential cells (LPTCs) that are clearly direction-selective^27^, cSIFE responds strongly to moving sinusoidal gratings, regardless of the direction of motion (Fig. 1a, *N*=16)^26^. cSIFE is also inhibited by stationary gratings, regardless of their orientation (Fig. 1b, *N*=16)^26^.

Natural scenes have greater spatial complexity than single-frequency sinusoidal gratings do. What is cSIFE's response to natural images? When the hoverfly views a stationary natural scene (Fig. 1c) cSIFE's spontaneous rate is also inhibited (Figs 1d, *n*=1), just like it was in response to sinusoidal gratings (Fig. 1b). To investigate the inhibition by stationary images in more detail we use both natural and artificial images. The images (Fig. 1c) have been used previously to investigate the responses of higher order visual neurons in the hoverfly, and are known to strongly stimulate the LPTCs that code for directional motion^25^,^28^,^29^,^30^. We find that cSIFE is inhibited by most of these six images too, but that the level of inhibition varies between them (Fig. 1e, *N*=16).

### cSIFE's inhibition depends on the slope constant

**Figure 2:**
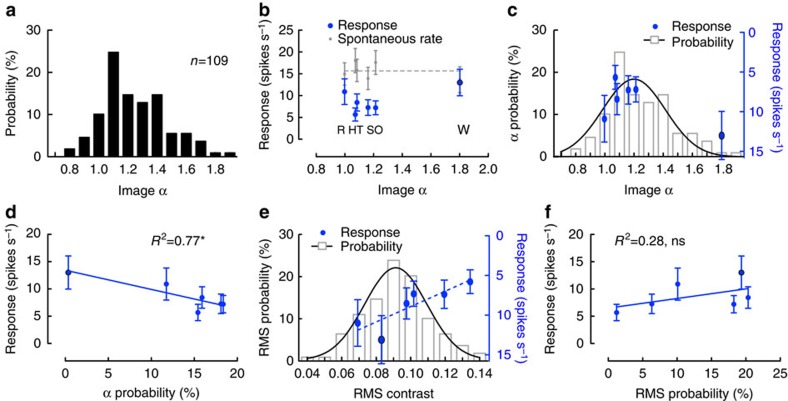
cSIFE's inhibition by natural images depends on the slope constant. (**a**) The distribution of *α*'s in 104 natural scenes from (tabby.vision.mcgill.ca/html/LandWater1.html)^33^, and the five natural images used in this study. (**b**) The spontaneous rate (grey) and the response (blue) to six natural scenes, as a function of their slope constant (Image α); *N*=16. The data are replotted from Fig. 1e. The dashed line shows the average spontaneous rate. (**c**) The distribution of image α's from **a** (grey), together with a Gaussian curve fit (black) to the distribution. The blue data are replotted from **b**, but the (right) *y* axis has been inverted and the baseline set to the average spontaneous rate (dashed line in **b**). (**d**) The cSIFE inhibition (replotted from **b**) as a function of the probability of its image α being present in a population of natural images (extracted from the Gaussian curve fit in **c**). Pearson correlation coefficient indicated, with a star (*) for *P*<0.05. (**e**) The distribution of effective RMS contrasts (grey) of the 109 images, after they have been bandpass filtered between 0.06 and 1 c.p.d., together with a Gaussian curve fit (black). The response to the six scenes, as a function of their effective RMS contrast in blue, *N*=16. The data are replotted from **b**, but inverted and with the baseline set to the mean spontaneous rate. (**f**) The cSIFE response during inhibition (replotted from **e**) as a function of the probability of the effective RMS contrast being present in a population of natural images (from the Gaussian curve fit in **e**). Pearson correlation coefficient indicated, ns. In **b**–**f** the response to the random image has a thin black line around its data point, and all error bars show s.e.m. ns, not significant.

Across natural scenes, the slope constants (α in Eq. 1) show a broad Gaussian distribution with a peak around 1–1.2 (refs 1, 11, 15, 31). However, hoverfly compound eyes have a limited spatial resolution, with maximal resolution of around 1 degree^32^. Furthermore, cSIFE's inhibition is not only limited by the spatial resolution of the eye, but is confined to a specific bandwidth of spatial frequencies between 0.06 and 1 c.p.d.^26^. Therefore, to get a more realistic account of the amplitude slope constants that are relevant for cSIFE's inhibition we calculate the slope constants of the amplitude spectra of 109 natural images using linear curve fitting between 0.06 and 1 c.p.d. (Fig. 2a). The images come from a published database (tabby.vision.mcgill.ca/html/LandWater1.html)^33^ and also include the natural scenes used here (Fig. 1c). A Gaussian curve fit to the slope constants show that the peak is found when this is 1.2 (image α, Fig. 2c), similar to previous descriptions^1^,^11^,^15^.

The data in Figure 1e show that cSIFE's inhibition varies between different natural scenes. Is it possible that this variation is a consequence of the different slope constants of the natural scenes, to thus give a better match to image statistics typically encountered? To test this hypothesis, we plot cSIFE's inhibition to the natural scenes as a function of their slope constants (fitted between 0.06 and 1 c.p.d.), and see that the peak inhibition is found at a slope constant ∼1–1.2 (Fig. 2b, same data as in Figure 1e). This suggests that cSIFE's inhibition is tuned to the spatial frequency spectrum of natural scenes.

To investigate this potential correlation in more detail we replot the distribution of slope constants in natural scenes from Fig. 2a (grey histogram and black Gaussian curve fit, Fig. 2c) together with the cSIFE inhibition from Fig. 2b. To allow for more direct comparison between the slope constants and the neural response, we plot the inhibition data inverted (blue, Fig. 2c), and set the baseline at the average spontaneous rate (dashed line in Fig. 2b). The inhibition (blue, Fig. 2c) appears to closely follow the probability distribution of amplitude slope constants (black, Fig. 2c). To statistically verify this observation, we plot the response to each image as a function of the probability of the slope constants of the image being present in a natural scene (Fig. 2d). This analysis shows a high and significant correlation (Pearson correlation coefficient, *R*^*2*^=0.7682, *P*<0.05, *N*=16) between the slope constant probability and cSIFE's response (Fig. 2d). This suggests that cSIFE's inhibition is indeed tuned to the spatial frequency spectrum of natural scenes.

Increasing the contrast of a stationary sinusoidal grating increases cSIFE's inhibition^26^. Is the inhibition that we see (Fig. 2b–d) in response to natural scenes caused by contrast differences of the images? Image contrast can be measured in many different ways^34^. We here use the root-mean square (RMS) contrast since it is related to the Fourier coefficients of the image and it is a good predictor of human perception of contrast^13^. We bandpass filter the images between 0.06 and 1 c.p.d. before calculating the effective RMS contrast, to take the bandwidth sensitivity of cSIFE into account^26^. Like the slope constant (image α), RMS contrast shows a Gaussian distribution across natural scenes, with a peak at 0.09 (grey histogram and black Gaussian curve fit, Fig. 2e). cSIFE's inhibition does not follow the distribution of RMS contrasts (blue data, Fig. 2e), and there is a poor correlation between the probability of the contrast being present in a natural scene, and cSIFE's response (Fig. 2f, Pearson correlation coefficient, *R*^2^=0.2765, non-significant (ns), *N*=16). However, cSIFE's inhibition increases with the RMS contrast of the image (Fig. 2e, Pearson correlation coefficient, *R*^2^=0.7326, *P*<0.05, *N*=16).

### Inhibition by manipulated images with a slope constant of 1

**Figure 3:**
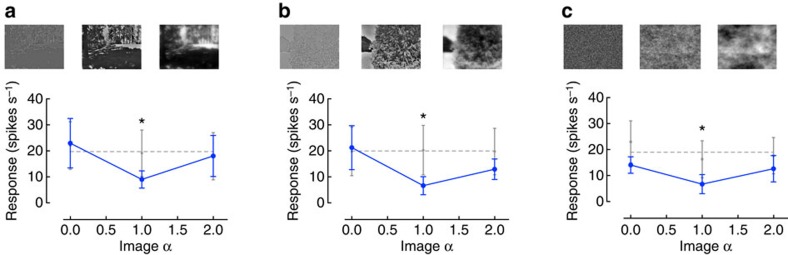
The inhibition to manipulated images confirm that inhibition depends on the slope constant. (**a**) The inhibition (blue) generated by the stationary Shadow image after manipulation of the amplitude spectrum. The insets show the reconstructed images at *α*=0, 1 and 2. Spontaneous rate in grey; *N*=5. (**b**) The inhibition generated by the Hill image at three different α's; *N*=5 (same neurons as in **a**). (**c**) The inhibition generated by the random image at three different *α*'s; *N*=5 (same neurons as in **a**,**b**). Significance was tested with a two-way ANOVA followed by Bonferroni's multiple comparisons test, *P*<0.05. All error bars show s.e.m.

The data in Figure 2 show that cSIFE's inhibition by stationary natural scenes follows the natural distribution of slope constants (Fig. 2d) and that it also increases with increasing RMS contrast (Fig. 2e). To investigate which of these two variables has the largest influence on cSIFE's inhibition, we create manipulated versions of the Shadow and the Hill images (Fig. 1c). If the first option is correct, and the level of inhibition is correlated with the slope constant, cSIFE's inhibition should decrease if we manipulate the slope constant (α) of an image away from 1. Indeed, we find that when the hoverfly is viewing the manipulated images, cSIFE is significantly inhibited when these have a slope constant of 1 (Fig. 3a, b, *N*=5, two-way analysis of variance (ANOVA) followed by Bonferroni's multiple comparison test, *P*<0.05), but not slope constants of 0 or 2 (Fig. 3a, b, *N*=5). Image slope constants of 0 and 2 are rarely found in natural scenes (Fig. 2a), and these images thus have highly artificial amplitude spectra.

Natural scenes have a non-random distribution of features^8^,^31^. To investigate how cSIFE's inhibition depends on the distribution of features we generate a new white noise image, with random phase and a flat amplitude spectrum (that is, a slope constant of 0). When the slope constant of the image is increased, the image is no longer ‘white', so we therefore refer to it as a random noise image. In response to this random noise image, cSIFE shows a similar dependence on the slope constant (image α), with a strong inhibition at a slope constant of 1 (Fig. 3c, *N*=5, two-way ANOVA followed by Bonferroni's multiple comparison test, *P*<0.05), but no difference to spontaneous rate at slope constants of 0 and 2.

**Figure 4:**
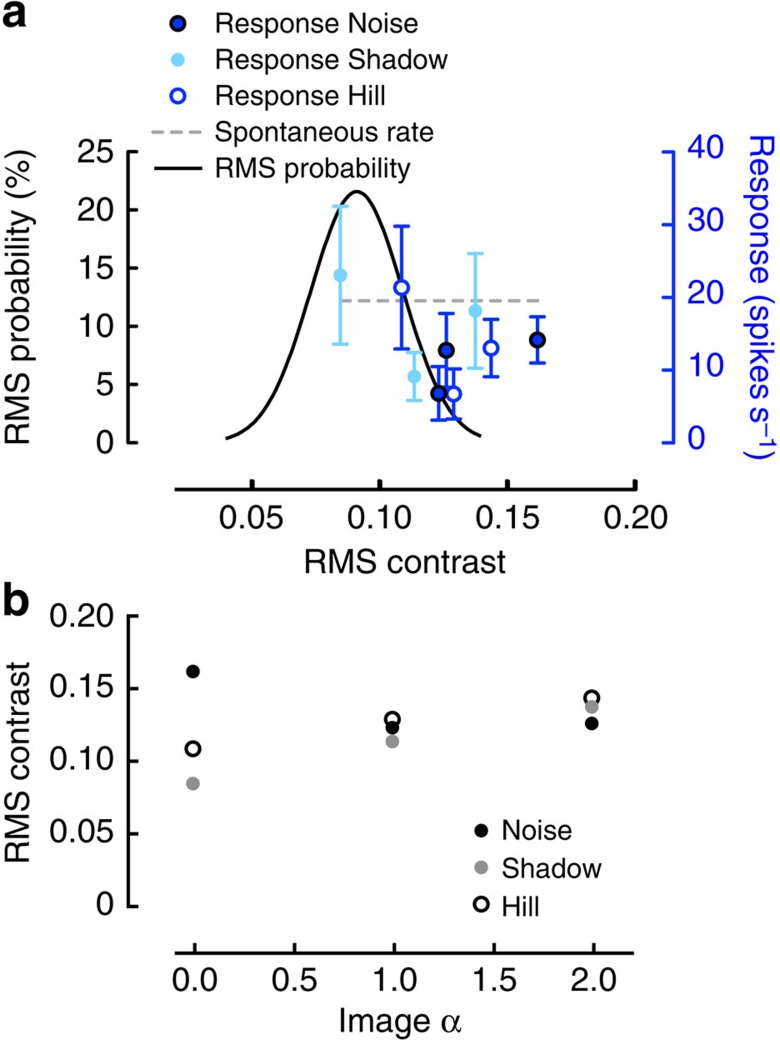
The inhibition does not depend on the effective RMS contrast. (**a**) The inhibition to the manipulated images, with the response plotted as a function of their effective RMS contrasts. The effective RMS contrasts were calculated after bandpass filtering the images to the relevant frequency spectrum for cSIFE. The data are replotted from Figure 3, *N*=5, with error bars showing s.e.m. The dashed grey line shows the average spontaneous rate. The solid black line shows the distribution of effective RMS contrasts in natural scenes, replotted from Figure 2e. (**b**) The RMS contrasts of the nine manipulated images as a function of their slope constants (image α).

To investigate the second option, we quantify whether the strong inhibition that we see at slope constants of 1 (Fig. 3) is an artefact of these images having the highest contrast. For this purpose we replot the data from Fig. 3, but now with the effective RMS contrast on the *x* axis. The resulting graph shows that cSIFE's inhibition does not increase with image contrast, but rather shows a scattered distribution (Fig. 4a). Neither is there a correlation between the effective RMS contrast probability distribution (histogram, Fig. 4a) and the cSIFE response (blue data, Fig. 4a). Furthermore, the data in Fig. 4b show the effective RMS contrasts of the nine images as a function of their slope constants. Despite the images with slope constants of 1 generating much stronger inhibition than those with a slope constant of 2 (Fig. 3), these images all have very similar RMS contrasts (Fig. 4b). Furthermore, the images with slope constants of 0 have very different RMS contrasts (Fig. 4b), despite none of them generating any inhibition (Fig. 3).

In summary, the data in Figs 3 and 4 show that the strong inhibition at α's close to 1 is more likely caused by a matching of the neural coding to naturalistic slope constants than by a dependence on effective RMS contrast. Furthermore, the data show that it is the slope constant that affects the inhibition, and not the phase of the image.

### Bandpass filtering tunes cSIFE to natural slope constants

The data above show that cSIFE's inhibition is selectively tuned to the 1/*f* statistics typical of natural scenes. In earlier work, van Hateren^16^ showed that neural low- and high-pass filters in the photoreceptors and LMCs improve responses to natural scenes with slope constants close to 1 by ‘whitening' the amplitude spectrum. We can use a similar approach to van Hateren^16^ (see also ref. 35) to investigate how cSIFE's selective spatial frequency tuning between 0.06 and 1 c.p.d.^26^ affects the response to the amplitude spectra of natural scenes.

**Figure 5:**
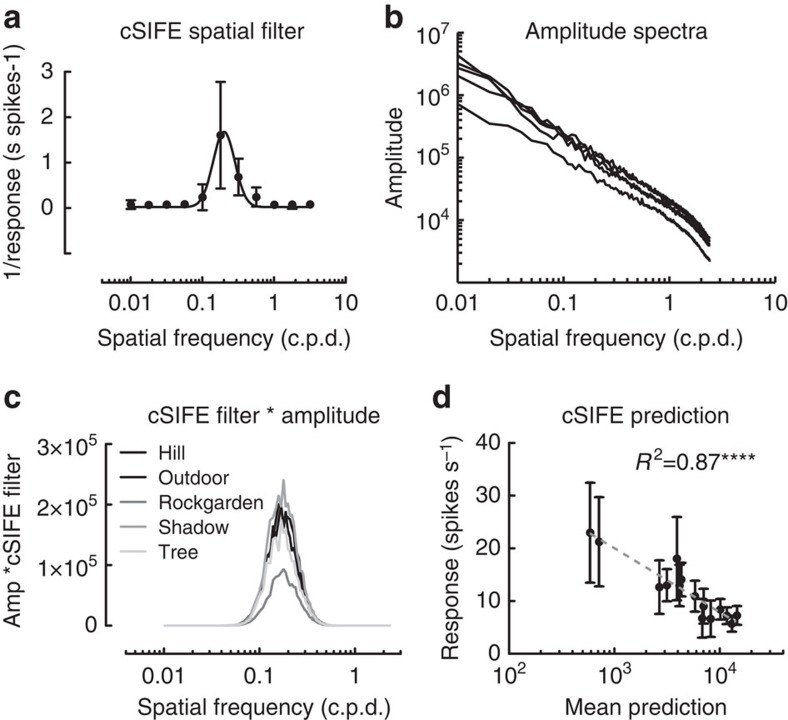
cSIFE's spatial frequency tuning predicts the response to natural scenes. (**a**) The spatial frequency tuning for cSIFE with the response inverted, so that stronger inhibition is plotted as an increase on the *y* axis. The data are replotted from ref. 26 with the error bars showing s.e.m. The black line shows a Gaussian curve fit to the spatial frequency tuning. (**b**) The amplitude spectra of the five natural scenes. (**c**) The spatial filter in **a** multiplied with the amplitude spectra in **b**. (**d**) The mean prediction (that is, cSIFE's spatial filter * the amplitude spectrum) for the 15 images used in this study, plotted against the neural response, with the error bars showing s.e.m. Pearson correlation coefficient indicated, *P*<0.0001.

For this purpose we first quantify cSIFE's spatial filter. We calculate the inverse response of cSIFE's spatial frequency tuning to stationary sinusoidal gratings^26^, to which we fit a log-normal function (which appears Gaussian, Fig. 5a). Note, however, that the published spatial frequency tuning data^26^ show inhibition for four data points only (Fig. 5a), so the Gaussian curve fit has to be taken with some caution, and the analysis below as preliminary. Nevertheless, as in (refs 16, 35) we then multiply the spatial filter (Fig. 5a), with the mean amplitude spectra (Fig. 5b shows the mean amplitude spectra for the five natural scenes in Figure 1c) to determine the output of cSIFE. This analysis suggests that the spatial tuning of cSIFE (Fig. 5a) amplifies the spectral energy of the images between 0.06 and 1 c.p.d. (Fig. 5c).

The data also show that the smallest output is generated to the Rockgarden image (Fig. 5c), which was indeed the image that generated the smallest inhibition (R, Fig. 1e). To investigate this potential correlation in more detail we plot the mean prediction (the integral of the spatial filter times the mean amplitude spectrum) for the 15 images used in this study, against the measured neural response. This graph shows that the mean prediction provides a good determinant of cSIFE's inhibition (Fig. 5d, Pearson correlation coefficient, *R*^2^=0.8654, *P*<0.0001 *N*=5 or 16). Our data thus suggest that the unique spatial frequency tuning of cSIFE^26^ may selectively enhance responses to the 1/*f* statistics typical of natural scenes, but this needs to be confirmed in future work.

### Behavioural responses to manipulated images

**Figure 6:**
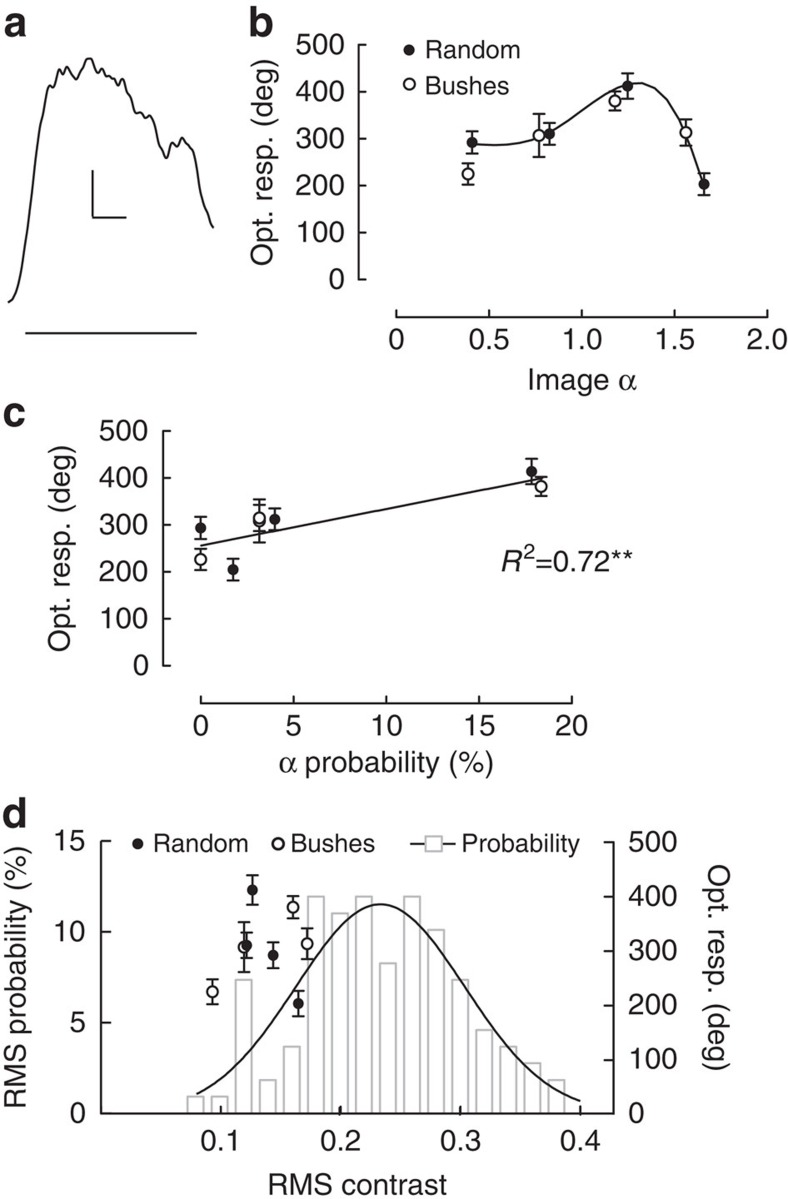
The hoverfly optomotor response depends on the slope constant. (**a**) The yaw optomotor response of hoverflies walking on a trackball setup. The random image (*α*=1.2) was moving at 110° s^−1^ and the behavioural yaw output digitized at 1 kHz (*N*=5, *n*=43). The line under the data shows the 10 s peri-stimulus duration. Scale bar shows 10 deg per second and 2 seconds. (**b**) The accumulated yaw optomotor response after 10 s stimulation with a natural (open symbols, Bushes^25^,^28^,^29^) or artificial (filled symbols) image manipulated to have different α values. (**c**) The accumulated optomotor response to the natural and random image (replotted from **c**) as a function of the probability of the *α* being present in a population of natural images (taken from the Gaussian curve fit in Fig. 2c). Pearson correlation coefficient indicated, *P*<0.01. (**d**) The distribution of effective RMS contrasts of the 109 images, after they have been low-pass filtered from 1 c.p.d. (grey), together with a Gaussian curve fit (black). The open and closed symbols show the optomotor response to the images (replotted from **a**). In **b**–**d** the error bars show the s.e.m.

In the experiments thus far we have quantified visual responses of the cSIFE neuron. Are hoverfly behavioural responses also affected by the slope constant of visual scenes? To investigate this, we measure the optomotor response of hoverflies walking on a trackball setup^36^,^37^. Trackball setups have been used previously to, for example, measure the optomotor response of flies^38^ and the optokinetic response of mice^39^. Here we see that when an image moves past the hoverfly at 110° s^−1^ the hoverfly tries to stabilize the optic flow by turning in the direction of the image motion (Fig. 6a, *N*=5, *n*=43).

For statistical analysis we quantify the accumulated yaw during 10 s of visual stimulation. This analysis shows that the strongest yaw optomotor response is generated by images with a slope constant around 1.2, whether the image is artificial (filled symbols, Fig. 6b, *N*=5, *n*=43–58) or natural (open symbols, Fig. 6b, *N*=2–6, *n*=28–71, two-way ANOVA shows a significant effect of image α, *P*<0.0001, but no significant difference between the two images). Above, we showed that the neuronal responses were correlated with the probability of that slope constant (α) being present in natural scenes (Fig. 2d). Similarly, in behaviour, the strength of the optomotor response is correlated with the probability of that image α being present in natural scenes (Fig. 6c, *P*<0.01). These data (Fig. 6b, c) thus suggest that the optomotor response is also tuned to the 1/*f* amplitude spectra of natural scenes.

Like neural responses to visual stimuli, behavioural responses depend on image contrast. The natural images that we use here are spatially filtered through the coarse optics of the hoverfly eye, with a maximal resolution of ∼1 degree^32^, thus working as a low-pass filter with a cut-off frequency of 1 c.p.d. (ref. 16). Therefore, we low-pass filter the images before quantifying the effective RMS contrast relevant for behaviour. The RMS contrast of 109 low-pass filtered natural scenes also show a Gaussian distribution, but with a peak at a contrast of 0.23 (grey histogram and black Gaussian curve fit, Fig. 6d), compared with 0.09 when the images were bandpass filtered (Fig. 2e). We find that there is no correlation between the RMS contrast of the images used in the trackball experiments and the optomotor response, nor between the optomotor response and the distribution of contrasts (Fig. 6d). We thus conclude that in response to these images, the optomotor response is affected by image α (Fig. 6b, c), and that this effect is not a consequence of the contrast of the images (Fig. 6d).

## Discussion

Fourier domain analyses of photographs of natural scenes show that the amplitude has a characteristic fall-off with spatial frequency, with slope constants close to 1 (ref. 3). Our data here show that the slope constant influences the level of inhibition generated in cSIFE, with peak inhibition at slope constants that are most similar to those of natural scenes (Figs 2). Such tuning to average spatial statistics would increase the efficiency with which the information of natural scenes can be processed, which is important since neurons have an inherently limited capacity to process information.

When amplitude spectra of natural scenes have slope constants of exactly 1, they are scale invariant, so that equal energy is found in each octave (1–2, 2–4 and 4–8 c.p.d. and so on)^4^. Field^31^ showed that the receptive fields of the mammalian primary visual cortex are arranged in a similar way, with increasing bandwidth with increasing spatial frequency, thus producing the most efficient coding scheme for scale-invariant natural scenes. Barlow^14^ suggested that the visual system should reduce redundancy by not coding the predictable parts of a signal^15^,^22^, and that the mammalian visual system is efficient because it is well matched to the statistical redundancy of the visual environment^23^,^31^. Indeed, psychophysical studies show that the output of the visual system is tuned to the amplitude spectra of natural scenes^11^,^40^,^41^,^42^.

A complementary view to the theory that the role of early visual processing is to reduce redundancy^14^,^17^ is that it maximizes information transmission^19^. By increasing redundancy, the visual system generates a more reliable signal-to-noise ratio, and thus a maximization of the amount of information that the central nervous system receives^12^. Van Hateren^19^ further showed that retinal filters that maximize information transmission actually reduce redundancy at high signal-to-noise ratios, but they simultaneously increase redundancy (and thus information transmission) at low signal-to-noise ratios.

cSIFE is a higher order neuron, which gets its input from peripheral photoreceptors and LMCs, which optimize the coding of natural scenes by being closely tuned to the average image statistics^2^,^12^,^16^,^35^. At high light levels such peripheral filters act as bandpass filters, and they are thus likely to contribute to the tuning of cSIFE that we describe here. However, to determine the contribution of such peripheral filters to the spatial frequency tuning of cSIFE, and whether additional processing takes place^26^, more work is needed. First, we need to measure and model the spatial frequency responses of the peripheral filters in *Eristalis*, under the light conditions used here, to, for example, determine the spatial extent of the lateral inhibition, which typically takes place between neighbouring lamina cartridges^17^,^43^,^44^. Second, this peripheral processing needs to be compared with the spatial frequency tuning of cSIFE, but with higher resolution: the curve fit in Figure 5a shows inhibition at only four spatial frequencies and must thus be viewed as preliminary.

**Figure 7:**
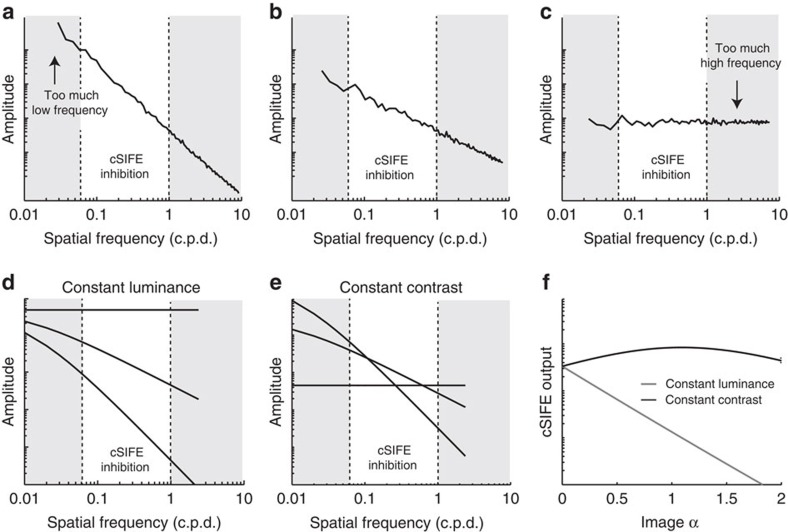
The influence of the slope constant on cSIFE's inhibition. (**a**) The graph shows the amplitude as a function of spatial frequency of an image with a slope constant of 2. The grey shaded areas show spatial frequencies where cSIFE is not strongly inhibited, whereas the white area shows the 0.06–1 c.p.d. range that generates strong inhibition. (**b**) The amplitude of an image with a slope constant of 1. (**c**) The amplitude of an image with a slope constant of 0. (**d**) The graph shows the amplitude as a function of spatial frequency of a filtered Noise image with a slope constant of 0, 1 or 2, where the luminance was held constant. (**e**) The graph shows the amplitude spectrum of the Noise image with a slope constant of 0, 1 or 2, where the bandpass filtered RMS contrast was held constant. (**f**) The predicted cSIFE output, that is, cSIFE's spatial filter multiplied with the amplitude spectra for the two conditions (in **d**,**e**).

Nevertheless, the model in Figure 5 suggests that cSIFE's spatial frequency tuning creates the highest inhibition to images with 1/*f* statistics typical of natural scenes, which can be explained with a simple diagram (Fig. 7). When an image's slope constant is high its spatial frequency spectrum rolls off steeply (Fig. 7a). Therefore, in images with high slope constants, the highest amplitude is found at low spatial frequencies, where cSIFE's inhibition is weak (grey shaded area, Fig. 7a). Reducing an image's slope constant reduces the amplitude at lower frequencies while increasing the amplitude at higher spatial frequencies. When the slope constant is close to 1 (Fig. 7b), more of the amplitude is found at intermediate spatial frequencies, where cSIFE is inhibited (white area, Fig. 7b). When the slope constant is decreased even further, more amplitude is found at high spatial frequencies (Fig. 7c), where cSIFE's inhibition is weak (grey shaded area, Fig. 7c), so total inhibition decreases. For cSIFE it thus seems that the optimal balance between low and high spatial frequencies is found at a slope constant close to 1. This hypothesis should be tested in future work by selectively manipulating the alpha in different frequency bands.

When changing one image parameter, other parameters change too. For example, if we change the slope constant of an image while keeping its total luminance constant (Fig. 7d), the average amplitude spectra look quite different compared with when we change the slope constant of an image while keeping its contrast constant (Fig. 7e). Using our model (Fig. 5), we see maximum inhibition to images with a slope constant close to 1 when the contrast is fixed, but when the luminance is fixed maximum inhibition is generated by images with a slope constant of 0 (Fig. 7f). This means that we can verify the model (Figs 5) experimentally by recording cSIFE responses to images with varied slope constants but other parameters fixed. Note, however, that the models presented here only assume spatial filters, and ignore temporal adaptation to, for example, prevailing luminance conditions (see, for example (ref. 28, 45)).

Previous work on cSIFE^26^ and other visual neurons in flies^46^,^47^ and mammals^48^ show a strong, nonlinear dependence on image contrast. However, most of those experiments used sinusoidal gratings or other experimenter-defined stimuli. Here we showed that cSIFE's inhibition increased with increasing effective RMS contrast in the unmanipulated natural scenes (Fig. 2e). This would suggest that the strength of the inhibition could be caused by the contrast of the images, and not by the slope constants. However, there was no correlation between the inhibition and the effective RMS contrast in the manipulated scenes (Fig. 4b), but a clear dependence on image slope constant (Fig. 3). Neither in behavior could we see a correlation between the effective RMS contrast and the optomotor response (Fig. 6d), despite contrast previously being shown to affect fly optomotor responses^49^,^50^. This suggests that the natural scene responses that we have recorded depend more on the slope constant than on image contrast. Indeed, previous work investigating LPTC responses to natural scenes, showed that the velocity tuning is remarkably resilient to the contrast of the images^25^,^29^, despite contrast having a large effect on the LPTC response to sinusoidal gratings^46^,^47^. It is thus non-trivial to directly compare the response dependence on image contrast between simple experimenter-designed stimuli and more naturalistic images.

Psychophysics show that the output of the human visual system is tuned to the amplitude spectra of natural scenes^40^,^41^,^51^. Our finding that the behavioural optomotor response of hoverflies is tuned to the slope constants typical of natural scenes provides further evidence for analogy between the human and insect visual systems. In human observers, different spatial frequencies serve different roles, so that for example low spatial frequencies are used for quick scene categorization^6^. If the *α* of an image is artificially increased, the resulting image appears to human observers as more blurry. Similarly, in a photo a higher *α* is typically induced by features that are blurry, either because they are out of focus, or because they were moving during the exposure time^4^. Human observers are very good at predicting the correct α of previously unseen natural images^4^. However, some scenes that are not perceived by human observers as blurry have inherently high *α*'s. These include closeup photos of natural objects such as flowers and leaves, and human faces and portraits^52^.

Our description of the responses of a single higher order visual neuron and a behavioural output that match the spatial statistics of natural scenes provide an example of striking similarity between higher order neural processing in the mammalian and invertebrate visual systems, similar to what has previously been shown for peripheral visual processing^53^,^54^,^55^. Despite having vastly different optics^56^, and phototransduction mechanisms^57^, flies and mammals appear to share the neural processing of natural scenes (see also^24^,^58^,^59^).

## Methods

### Images

For analysis of image statistics we used 104 landscape images from a public library (http://tabby.vision.mcgill.ca/html/LandWater1.html)^33^ and images previously used by us^25^. In electrophysiology we selected five naturalistic images from a larger dataset of over 20 images used previously in investigations of motion vision responses in the hoverfly^25^,^28^,^29^. In addition, we selected a filtered random noise image^30^, which has also been used previously to investigate motion vision in hoverflies. Since we originally believed that contrast was the determining factor, we opted to use a random noise image with an RMS contrast of typical natural scenes (see Fig. 2e). In addition, we generated a white noise texture in Matlab by assigning each pixel in a 480 × 640 matrix a pseudo-random value from the uniform distribution and linearly rescaling the pixels from 0 to 255. For image analysis we assumed that the images from the database were 100° wide. For the images used in the experiments we calculated the subtense as seen by the hoverfly during experiments, and quantified image data for the part of the image seen by the hoverfly.

To calculate the distribution of slope constants (*α*'s) across the images (*n*=109), we converted them to greyscale and used a Fourier transform to extract the amplitude spectrum (for step-by-step guides, see Supplementary Methods and Supplementary Figure 1). We quantified the average amplitude across all orientations as a function of spatial frequency, and plotted this on a log–log scale. The slope constant of the amplitude spectrum (image α) was identified by fitting a linear function to the average amplitude spectrum between 0.06 and 1 c.p.d. (similar to refs 4, 9, 11).

We calculated the RMS contrast^13^,^34^ using the function:


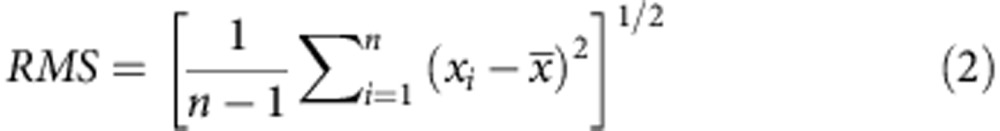


where *n* is the number of pixels, x_*i*_ is a normalized grey level value between 0 and 1 and 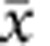 is a mean normalized grey level:


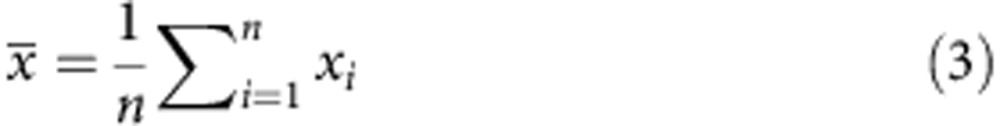


Before calculating the RMS contrast we bandpass filtered the images between 0.06 and 1 c.p.d. to take the sensitivity of cSIFE into account^26^, or low-pass filtered the images from 1 c.p.d. to take the optics into account^32^. The RMS contrasts of the bandpass filtered images were used to analyse electrophysiology responses and the low-pass filtered images to analyse behavioural responses. Table 1 shows the slope constants (*α*'s) and RMS contrasts for all images used in the experiments.

We manipulated the slope constants as described in Tolhurst and Tadmor^41^. Briefly, we first converted each image to greyscale (for step-by-step guides, see Supplementary Methods). Then we performed a two-dimensional Fourier transform and calculated the amplitude spectrum, which is the orientation-averaged amplitude as a function of spatial frequency. We then divided the Fourier-transformed image by its amplitude spectrum to get a flat one, with an *α* of 0. By multiplying the result with the coefficient (1+ k*f^*-α*^), where k is a constant, we could generate any desired image α. By then doing an inverse Fourier transform and rescaling the image matrix from 0 to 255, we recreated the images, but now with a different α.

### Electrophysiology

*Eristalis tenax* larvae were collected from cow dung at Cederholms Lantbruk. The larvae were brought to the laboratory to pupate and hatch in a 12:12 h light:dark cycle at ∼22 °C. After hatching, adult flies were stored in a fridge (at 5 °C). Twice a week the hoverflies were brought to room temperature and fed *ad libitum* with pollen, honey and water. At experimental time the hoverfly was immobilized with a bee wax and resin mixture. The head was tilted forward and a hole cut over the left lobula plate. The fly was placed 12–13 cm in front of a linearized CRT monitor with a temporal resolution of 160 Hz and a spatial resolution of 640 × 480 pixels, corresponding to ∼100 × 75 degrees of the fly's field of view. Visual stimuli were displayed using Flyfly (www.flyfly.se) and the psychophysics toolbox (psychtoolbox.org) in Matlab (www.mathworks.com).

We recorded intracellular responses using sharp alumino silicate electrodes pulled on a P-1000 Brown–Flaming electrode puller (Sutter instruments, San Francisco). Data were amplified with a BA-03X amplifier (NPI electronics, Germany) and 50 Hz noise reduced with a Humbug (Quest Scientific, Canada). The data were acquired and digitized at 10 kHz using a NiDAQ 16 bit data acquisition card (NI USB-6210, National Instruments) and the data acquisition toolbox in Matlab. cSIFE neurons were identified based on their non-directional excitation to the motion of a sinusoidal grating (8°, 5 Hz, Fig. 1a, *N*=16) and their inhibition to the same gratings when stationary (8°, 0 Hz, Fig. 1b, *N*=16)^26^.

Data were analysed using Matlab. The spontaneous rate was calculated for 500 ms pre-stimulus onset (‘spont', Fig. 1d). Response inhibition was calculated for 780 ms starting 180 ms post-stimulus onset (‘inhib', Fig. 1d). Repetitions (*n*) within one neuron were averaged before averaging across animals (*N*). All responses are shown as average number of action potentials per second, with error bars indicating s.e.m.

### Behaviour

To investigate the optomotor response we used a trackball setup as described previously^60^. Two optical sensors (extracted from Razer Imperator ergonomic gaming mice, Razer Inc) provided information about the ball's motion (for equations see, for example,^61^) and our in-house Flytracker software written in Matlab digitized the data at 1 kHz for offline analysis. Wing-fixed, tethered *E. tenax* hoverflies were placed on the air supported trackball (a 1.45 g styrofoam ball, 50 mm diameter), 8 cm in front of the CRT screen. During each trial a panorama rotated at 110° s^−1^ for 10 s. We used two panoramas: Bushes^25^,^28^,^29^, and the random noise image described above. Between trials the screen was left at mid luminance for a minimum of 2 s.

We quantified the accumulated yaw walked during the 10 s of stimulus motion. Before averaging the accumulated yaw across trials, we removed statistical outliers, defined as trials where the response deviated more than 2 s.d. from the mean. The data in the figures show the mean across trials (*n*)±s.e.m., where *N*=5 for the Random image and *N*=5 for *α*=0.4 and 1.6; *N*=2 for *α*=0.8 and *N*=6 for *α*=1.2 for the Bushes image.

### Statistics

Statistical analysis was done using Graphpad Prism software (La Jolla, CA, USA). For statistical analysis of significance we performed two-way ANOVAs, followed by Bonferroni correction for multiple comparisons, with significance set to *P*<0.05.

We quantified the frequency distribution of image parameters for the 109 images using the histogram function in Prism. We then fitted the probability distribution with a Gaussian function using least squares optimization:


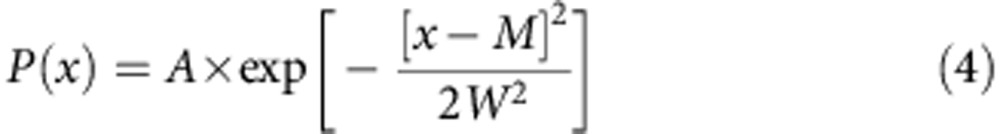


Where *P* is the probability, *A* the amplitude of the function, *M* its peak position and W defines the width of the function. From the Gaussian function we could extract the probability measure of a parameter (that is, *α* or effective RMS contrast) being present in a natural scene. The probability (*P*) values for the images used in recordings were then correlated with the biological response to the same image using Pearson's product-moment correlation.

## Additional information

**How to cite this article:** Dyakova, O. *et al*. A higher order visual neuron tuned to the spatial amplitude spectra of natural scenes. *Nat. Commun.* 6:8522 doi: 10.1038/ncomms9522 (2015).

## Supplementary Material

Supplementary Information Supplementary Figure 1 and Supplementary Methods

## Figures and Tables

**Table 1:** Image slope constants and contrasts.

**Figure**	**Image**	α	**Contrast (electrophysiology)**	**Contrast (behaviour)**
1, 2, 5	Hill	1.0731	0.1346	
1, 2, 5	Outdoor	1.2142	0.1018	
1, 2, 5	Rockgarden	0.9978	0.0692	
1, 2, 5	Shadow	1.1619	0.1196	
1, 2, 5	Tree	1.0845	0.0976	
1–2	Random noise	1.8024	0.0830	
3–4	Random noise	0.0001	0.1618	
3–4	Random noise	1.0000	0.1232	
3–4	Random noise	1.9982	0.1261	
3–4	Hill	0.0003	0.1086	
3–4	Hill	1.0000	0.1290	
3–4	Hill	1.9994	0.1437	
3–4	Shadow	−0.0006	0.0846	
3–4	Shadow	1.0000	0.1136	
3–4	Shadow	1.9993	0.1375	
6	Random panorama	0.4110		0.1428
6	Random panorama	0.8276		0.1208
6	Random panorama	1.2505		0.1255
6	Random panorama	1.6625		0.1637
6	Bushes panorama	0.3881		0.0924
6	Bushes panorama	0.7719		0.1186
6	Bushes panorama	1.1820		0.1592
6	Bushes panorama	1.5644		0.1710

RMS, root-mean square.

The data show the slope constants (α's) and the effective RMS contrasts of the images used in the study. For analysis we used the part of the image seen by the hoverfly. The slope constant (image α) was calculated by polynomial fitting between 0.06–1 c.p.d. The effective RMS contrast for the images used in electrophysiology was calculated after bandpass filtering the images between 0.06–1 c.p.d. The effective RMS contrast for the images used in behaviour was calculated after low-pass filtering the images from 1 c.p.d.
