# Supplementary material for: A higher order visual neuron tuned to the spatial amplitude spectra of natural scenes
Source: Nat Commun. 2015 Oct 6;6:8522. doi: 10.1038/ncomms9522 (PMC4600736; doi:10.1038/ncomms9522)
Supplement: Supplementary Information — Supplementary Figure 1 and Supplementary Methods [file ncomms9522-s1.pdf]

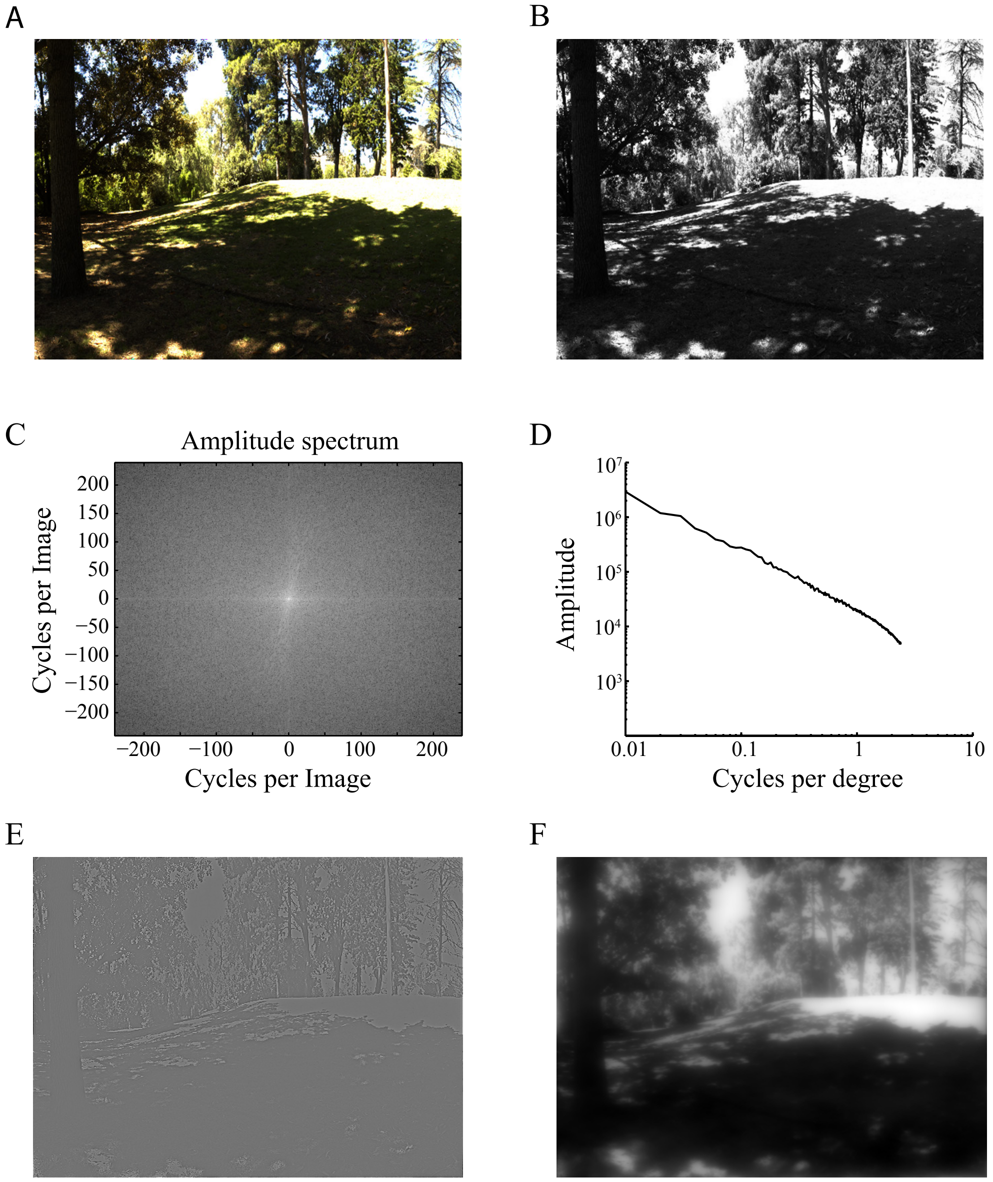

Supplementary Figure 1. Image manipulation method.
A) For image manipulation we started with color images. The panel displays the part of the 'Shadow' image used in electrophysiology. B) The grayscale version of the same image. C) The amplitude spectrum of the image in panel B. D) The line shows the rotationally averaged amplitude spectrum of the image in panel B, which has a slope constant of 1.16. E) The Shadow image with an α of 0. F) The Shadow image with an α of 2.

Supplementary methods

1. To calculate the alpha of an image:
   1.1. Read the image (Supplementary Figure 1A), using Matlab's function *imread.*
   1.2. If relevant, convert the image to greyscale (Supplementary Figure 1B) using Matlab's function *rgb2gray.*
   1.3. Use the supplied function ***get_alpha.m*** to perform the following steps:
      1.3.1. Perform a Fourier transformation of the greyscale image (using Matlab's function *fft2*).
      1.3.2. In the output of the Fourier transformation, move the zero-frequency component to the center (using Matlab's function *fftshift*).
      1.3.3. Extract the amplitude spectrum (Supplementary Figure 1C) using the following equation:

$$A(z) = \left[(\mathrm{Re}\, z)^2 + (\mathrm{Im}\, z)^2 \right]^{1/2}$$

where *Re* and *Im* are the real and imaginary part of complex number *z*, respectively. The equation utilizes Matlab's function *abs*.

      1.3.4. Get the polar coordinates (using Matlab's function *cart2pol*) and calculate the average amplitude spectrum across all orientations.
      1.3.5. Calculate the rotational average of the amplitude spectrum (Supplementary Figure 1D).
      1.3.6. Perform polynomial data fitting and extract the slope constant (α) as a first coefficient (using Matlab's function *polyfit*). We did the fitting between 0.06 and 1 cycles per degree (cpd).
      1.3.7. Plot the rotationally averaged amplitude as a function of spatial frequency, both on logarithmic scales.
2. To change the slope constant of the average amplitude spectrum (α):
   2.1. Use the supplied function ***change_slope.m***, to perform the following:
      2.1.1. Read the image (Supplementary Figure 1A), using Matlab's function *imread.*
      2.1.2. If relevant, convert the image to greyscale (Supplementary Figure 1B) using Matlab's function *rgb2gray.*
      2.1.3. If wanted, resize the image using Matlab's function *imresize.*

2.1.4. Perform a Fourier transformation of the greyscale image (using Matlab's function *fft2*).

2.1.5. In the output of Fourier transformation, move the zero-frequency component to the center (using Matlab's function *fftshift*).

2.1.6. Extract the amplitude spectrum (Supplementary Figure 1C) using the following equation:

$$A(z) = \left[(\mathrm{Re}\,z)^2 + (\mathrm{Im}\,z)^2\right]^{1/2}$$

where *Re* and *Im* are the real and imaginary part of complex number *z*, respectively. The equation utilizes Matlab's function *abs*.

2.1.7. Get the polar coordinates (using Matlab's function *cart2pol*) and calculate the average amplitude spectrum across all orientations.

2.1.8. Calculate the rotational average of the amplitude spectrum.

2.1.9. Set the slope constant to "zero" by dividing the Fourier matrix by the average amplitude spectrum.

2.1.10. Set the slope constant (α) to any desired value (Supplementary Figure 1D) by multiplying the result with the coefficient f^{α}.

2.1.11. Move the zero-frequency component back from the center (using Matlab's function *ifftshift*).

2.1.12. Perform an inverse Fourier transformation using Matlab's function *ifft2*.

2.2. Resize the image if this is needed for experiments (using Matlab's function *imresize*).

2.3. We rescaled the new image to get luminance values between 0 to 255 using:

$$I_{0-255} = \frac{I_i - I_{\min}}{I_{\max} - I_{\min}} \times 255$$

where I_i represents each pixel of an image, and I_{min} and I_{max} the minimum and maximum image luminance. The appropriate rescaling should be set to optimize the use of the range of the screen used for visual display (Supplementary Figure 1E, F).

3. <u>To change the slope constant (α) for panoramas:</u>

3.1. Split the panorama in N equal parts and save them as new images (we used N=12 and N=13).

3.2. Perform steps 2.1.1-2.1.12 from *“To change the slope...”* for each of new image from step 1.

3.3. Create new panoramas from the patches with modified α’s, using Adobe Photoshop’s *photomerge* function.

4. <u>To calculate the effective RMS contrast:</u>

4.1. Read the image, using Matlab’s function *imread.*

4.2. Rescale the image to the greyscale if relevant (using Matlab’s function *rgb2gray).*

4.3. We bandpass filtered or lowpass filtered the images before performing further steps:

4.3.1. Bandpass filtering was done using our function ***bandpass_imfilt.m****,* with a Butterworth bandpass filter.

4.3.2. Lowpass filtering was done using our function ***lowpass_imfilt.m****,* with a first order Butterworth lowpass filter.

4.4. Use our function ***getrms.m*** to:

4.4.1. Return the filtered image with double precision (using Matlab’s function *double*).

4.4.2. Get the RMS contrast using the equation:

$$RMS = \left[\frac{1}{n-1} \sum_{i=1}^{n} (x_i - \bar{x})^2 \right]^{1/2}$$

```
function alpha=get_alpha(image,f1,f2)

% This function calculates the slope of the average amplitude
% spectrum across all orientations, and plots the output on a loglog scale.
%
% Input:
% image: name of a NxM matrix representing an image (any image can be read
% into this matrix using matlab's function "imread". The image
% can be rescaled to gray scale with matlab's "rgb2gray" function).
%
% f1, f2 - frequency band between which fitting of the amplitude spectrum will
% be done. The frequencies should be given in cycles per image.
%
% Olga Dyakova, Uppsala University, Motion Vision Group, 2014
% ref. Bruno Olshausen function "rotavg", available online
% at http://redwood.berkeley.edu/bruno/ VS212B/ lab2/rotavg.m
% and Dr Cyril Pernet, Fourier Analysis for neuroscientists A practical
% guide using Matlab: http://www.sbirc.ed.ac.uk/cyril/download/DTP_Fourier_analyses.pdf


fft_image = fftshift(fft2(image));%perform Fourier transform and
%move the zero-frequency component to the center
abs_im=abs(fft_image);% get the amplitude spectrum
[N, M]=size(abs_im);
% find the rotational average
[X, Y]=meshgrid(-M/2:M/2-1,-N/2:N/2-1);
[~, rho]=cart2pol(X,Y); %get the polar coordinates
rho=round(rho);
abs_av=nan(1,N/2);
for r=0:N/2-1
  idx= rho==r;
  abs_av(r+1)=mean(abs_im(idx));%get the rotationally averaged ampl. spectrum
end

freq=0:N/2-1;
figure
loglog(freq,abs_av,'k') % plot the average amplitude spectrum on loglog scale
xlabel('spatial frequency')
ylabel('average amplitude spectrum')
xx=log(freq(f1:f2));         % cut low and high frequencies
yy=log(abs_av(freq(f1:f2))); % get rotationally averaged ampl. spectrum
                             % data points for fitting

p=polyfit(xx,yy,1); % perform fitting
alpha=(-1)*p(1); % get the alpha
display(alpha);
```

```
function f=change_slope(original_image, alpha, square, gray, new_file_name)

% This function reads an image, and creates and saves a new image with any
% given alpha (i.e. the slope constant).
%
% Input:
% original_image - file name of the starting image, give the name as e.g.: ('name.png')
%
% alpha - the required alpha, as a positive number,
% (with increasing alpha the new image will be perceived as more blurry)
%
% square: 0 (if the image is not a square (MxM)) or 1 (if the image is a square)
%
% gray: 0 (if image is not in grayscale) or 1 (if the image is in grayscale)
%
% new_file_name - name of the new image
%
% Olga Dyakova, Uppsala University, Motion Vision Group, 2013

image=imread(original_image); %read image
if gray==0
    image=rgb2gray(image);% convert rgb image to grayscale
end
L=min(size(image)); % find the minimum width or height
if square==0
    image=imresize(image, [L L] ,'bicubic'); % resize image to a square
end
fft_image=fftshift(fft2(image)); % perform a Fourier transform and shift the
                                 % zero-frequency component to the center
                                 % of the output of the Fourier transformation
abs_im=abs(fft_image); %get an amplitude spectrum image
freq=-L/2:L/2-1; %set the spatial frequencies
[x, y]=meshgrid(freq,freq); % get a full grid of coordinates
[~, ro]=cart2pol(x,y); % transform data to polar coordinates
ro=round(ro);

%% get an average amplitude spectrum across all orientations
abs_im_av=zeros(L,L);
for r=0:L-1
    idx= ro==r;
    temp=mean(abs_im(idx));
    abs_im_av(idx)=temp;
end
%%
zeroslope=fft_image./((abs_im_av)); % set the flat amplitude spectrum
filter_image=zeroslope.*(1+ro).^(-alpha); % get the required slope
%% make new image
filter_image_shiftback=ifftshift(filter_image);
image_new=(real(((ifft2(filter_image_shiftback)))));
f=uint8(((image_new-min(image_new(:)))./((max(image_new(:))-min(image_new(:)))).*255);
%%
figure
imshow(f)
imwrite(f, new_file_name);
end
```

```
function image_new = bandpass_imfilt(image, d1, d2, n)

% This function filters an image with a Butterworth bandpass filter
%
% Input:
% image: name of a NxM matrix representing an image (any image can be read
% into this matrix using matlab's function "imread". The image
% must be rescaled to grayscale with matlab's "rgb2gray" function).
%
% d1, d2 - frequency limits of the bandpass filter, given in cycles per image
% n - filter's order
%
% output:
% filtered image
%
% Olga Dyakova, Motion Vision Group, Uppsala University, 2015

fft_image = fftshift(fft2(image));
[x,y] = size(image);
freq1=-x/2:x/2-1;
freq2=-y/2:y/2-1;
[x1,y1]=meshgrid(freq2,freq1);
[~, ro]=cart2pol(x1,y1);
ro1=round(ro);

filter=zeros(x,y);

for r1=0:x-1
    idx= ro1==r1;
    temp = (1/(1 + (r1/d2)^(2*n))).*(1-1/(1 + (r1/d1)^(2*n)));
    filter(idx)=temp;
end

imagefilt=fft_image.*filter;

filter_image_shiftback=ifftshift(imagefilt);
image_new=(real(((ifft2(filter_image_shiftback)))));
image_new=uint8(((image_new-min(image_new(:)))./((max(image_new(:)))-min(image_new(:)))).*255);
figure
imshow(image_new);
```

```
function image_new = lowpass_imfilt(image, d)

% This function filters an image with a Butterworth lowpass filter
% Input:
% image: name of a NxM matrix representing an image (any image can be read
% into this matrix using matlab's function "imread". The image
% must be rescaled to grayscale with matlab's "rgb2gray" function).
%
% d - cutoff frequency, given in cycles per image
%
% output:
% the filtered image
%
% Olga Dyakova, Motion Vision, 2015

[x,y] = size(image);
fft_image = fftshift(fft2(image));

freq1=-x/2:x/2-1;
freq2=-y/2:y/2-1;
[x1,y1]=meshgrid(freq2,freq1);
[~, ro]=cart2pol(x1,y1);
ro1=round(ro);
filter=zeros(x,y);

for r1=0:x-1
    idx= ro1==r1;
    temp=1/(1+(r1^2)/d^2);
    filter(idx)=temp;
end
imagefilt=fft_image.*filter;

filter_image_shiftback=ifftshift(imagefilt);
image_new=(real(((ifft2(filter_image_shiftback)))));
image_new=uint8(((image_new-min(image_new(:)))./((max(image_new(:)))-min(image_new(:)))).*255);
figure
imshow(image_new)
```

```
function rms_contr = getrms(image)

% This function calculates the RMS contrast of an image
%
% Input:
% image: name of a NxM matrix representing an image (any image can be read
% into this matrix using matlab's function "imread"). The image
% can be rescaled to grayscale with matlab's "rgb2gray" function).
%
% To filter the image before analysis use either
% bandpass_imfilt.m or lowpass_imfilt.m
%
% output: rms-contrast, scaled from 0 - 1
%
% Olga Dyakova, Motion Vision Group, Uppsala University, 2015

[n,m]=size(image);
im_new=double(image)/255;
im_mean=mean2(im_new);
under_sum=(im_new-im_mean).^2;
im_sum=sum(sum(under_sum));
rms_contr=sqrt((1/(n*m))*(im_sum));
```
